# The impact of an app-delivered mindfulness-based intervention on resting-state functional connectivity in pediatric concussion: a pilot study

**DOI:** 10.3389/fnins.2026.1771767

**Published:** 2026-09-17

**Authors:** Olivier Brown, Andra Smith, Molly Cairncross, Andrew B. Dodd, Gary S. Goldfield, Clare Gray, Ashley D. Harris, Natalia Jaworska, Bechara J. Saab, Veronik Sicard, Noah D. Silverberg, Lisa A. S. Walker, Roger Zemek, Andrée-Anne Ledoux

**Affiliations:** 1 Children’s Hospital of Eastern Ontario Research Institute, Ottawa, ON, Canada; 2 School of Psychology, University of Ottawa, Ottawa, ON, Canada; 3 University of Ottawa Brain and Mind Research Institute, University of Ottawa, Ottawa, ON, Canada; 4 Department of Psychology, Simon Fraser University, Burnaby, BC, Canada; 5 BC Children’s Hospital Research Institute, Vancouver, BC, Canada; 6 Rehabilitation Research Program, Centre for Aging SMART, Vancouver Coastal Health Research Institute, Vancouver, BC, Canada; 7 Department of Pediatrics, University of Ottawa, Ottawa, ON, Canada; 8 Department of Psychiatry, University of Ottawa, Ottawa, ON, Canada; 9 Department of Radiology, Hotchkiss Brain Institute, Alberta Children’s Hospital Research Institute, University of Calgary, Calgary, AB, Canada; 10 Department of Cellular Molecular Medicine, University of Ottawa, Ottawa, ON, Canada; 11 Royal Ottawa Mental Health Centre, Ottawa, ON, Canada; 12 Mobio Interactive Pte Ltd., Singapore, Singapore; 13 Department of Psychology, University of British Columbia, Vancouver, BC, Canada; 14 Ottawa Hospital Research Institute, Ottawa, ON, Canada

**Keywords:** concussion, fMRI, functional connectivity, mindfulness, pediatric

## Abstract

**Rationale:**

Concussion disrupts brain connectivity, and effective treatments remain limited. Mindfulness-based interventions (MBIs) may influence functional connectivity (FC) of networks affected by concussion, but pediatric trials are lacking. This study investigated whether an app-delivered MBI produced greater FC changes than a sham condition across the default mode (DMN), salience (SN), and central executive (CEN) networks.

**Methods:**

This randomized clinical trial (NCT05105802) recruited participants aged 12.00–17.99 years from the emergency department or interdisciplinary concussion clinic, then randomly assigned them to MBI or cognitive sham. Brain imaging sessions occurred at 72 h and 4 weeks post-concussion. Region-of-interest and seed-to-voxel analyses examined Group × Time interactions in FC across DMN, SN, and CEN (*p* < 0.050_*FDR*_).

**Results:**

Thirty-six participants were included (MBI: *n* = 16, median [IQR] age = 14.23 [13.52–15.65] years, 31% female; Sham: *n* = 20, median [IQR] age = 14.05 [13.13–16.26] years, 45% female). Intention-to-treat analyses revealed significant Group × Time interactions across networks. DMN-CEN FC patterns differed, with MBI participants showing diminished FC. Within DMN, four clusters showed significant interactions (*p* < 0.050_*FDR*_), three exhibiting larger FC reductions at 4 weeks in MBI participants. MBI participants showed greater FC increase in one SN cluster (*p* = 0.040_*FDR*_) and larger FC decrease in one CEN cluster (*p* = 0.024_*FDR*_) at 4 weeks. Per-protocol analyses identified one significant DMN cluster (*p* < 0.001_*FDR*_) where MBI participants exhibited greater FC increases.

**Conclusion:**

Mindfulness-based intervention training led to changes in FC in networks implicated in concussion recovery. Larger studies are needed to support these findings.

**Clinical trial registration:**

https://clinicaltrials.gov/study/NCT05105802, identifier NCT05105802.

## 1 Introduction

Approximately 10% of youth reported having a traumatic brain injury in the past year, with concussion being the most common type (Daugherty et al., 2024; McCrory et al., 2017; Taylor et al., 2017). Children and adolescents with acute concussion may experience a range of somatic, emotional, and cognitive symptoms that can persist for months to years, impacting quality of life (Beauchamp et al., 2019; McCrory et al., 2017; Novak et al., 2016; Yeates, 2010). Children and adolescents are at 40% increased risk of having a mental health problem within 10 years following a concussion (Ledoux et al., 2022). A scoping review found that increased emotional distress was associated with prolonged symptoms and that prolonged symptoms were strongly associated with depression (Sheldrake et al., 2022). Concussion can lead to alterations in brain structure and function, including changes in resting-state functional connectivity (FC), white matter integrity, cerebral perfusion, and neurovascular coupling (Churchill et al., 2017; Manning et al., 2017; Meier et al., 2020). Preventative, early rehabilitation programs, such as interventions focusing on building psychological resiliency, emotional regulation, and self-efficacy, might be key to managing concussions and promoting neural recovery, i.e., reversing concussion-related changes in the brain.

Concussion disrupts resting-state FC of key brain networks, including the default-mode network (DMN), salience network (SN), and central executive network (CEN), with context-dependent alterations characterized by both increases and decreases in connectivity. The DMN is implicated in self-referential processing, cognitive control, and emotional regulation (Frewen et al., 2020). The SN is implicated in processing salient stimuli and switching between DMN and CEN activation (Menon and Uddin, 2010). The CEN is implicated in cognition and working memory, influencing top-down processing of information (Seeley et al., 2007). Similar to anxiety and depression, increased FC within the DMN (Cheng et al., 2018; Coutinho et al., 2016; Li et al., 2023), a key network linked to rumination (Chou et al., 2023), was also found in concussion (Abbas et al., 2015; Newsome et al., 2016; Orr et al., 2016). In concussion, some reports indicate that the DMN changes are dynamic with acute phases of injury characterized by hyperconnectivity, such as increased FC between hubs and non-hubs within the DMN (Bouchard et al., 2024; Boshra et al., 2020), followed by a decrease in FC beyond the acute period (Bouchard et al., 2024; Zhu et al., 2015). FC within the DMN was found to decrease at 1 week post-injury compared to 1 day post-injury, with only partial recovery by 1 month post-injury (Zhu et al., 2015). The SN and CEN also demonstrated changes following concussion, with increased within- and between-network FC (Borich et al., 2015; Healey et al., 2022; Liu et al., 2020; Marusak et al., 2017; Orr et al., 2016; Stephenson et al., 2020). Such hyperconnectivity has been linked to persistent symptoms (Dunkley et al., 2018), suggesting that excessive network coupling may impede recovery. Psychological interventions that target these networks directly might be key in promoting neurophysiological recovery, restoring FC and improving emotional and cognitive outcomes throughout concussion recovery.

Mindfulness-based interventions (MBI) are evidence-based psychological treatments designed to cultivate mindfulness, a state of focused, nonjudgmental awareness of the present moment. MBIs often incorporate practices such as meditation, breathing exercises, and body awareness to enhance emotional regulation and promote adaptive coping strategies (Kabat-Zinn, 1990). In healthy youth, MBIs are generally associated with increased FC within and between the DMN, SN, and CEN (Bauer et al., 2020; Bremer et al., 2022; Hafeman et al., 2020; Jande et al., 2025; Mishra et al., 2020; Patsenko et al., 2019; Tang, 2017; Taren et al., 2017), reflecting context-sensitive moderation of resting-state network connectivity. However, in clinical populations characterized by network hyperconnectivity, MBIs were linked to normalization of connectivity patterns rather than uniform increases. For instance, in major depressive disorder, where DMN hyperconnectivity is implicated in rumination and maladaptive behavior, MBI participation was associated with reduced DMN FC, including decreased coupling between the rostral anterior cingulate cortex and the DMN, a change proposed as a mechanism underlying antidepressant effects (Sezer et al., 2022; Yang et al., 2016). The DMN, SN, and CEN are thought to function synergistically to facilitate self-regulatory processes, suggesting that the effects of MBIs on behavioral outcomes might be mediated by reorganization within and between these networks (Jande et al., 2025; Melis et al., 2022; Young et al., 2018). However, there is limited research directly examining the effects of MBI on these networks in pediatric populations in the context of concussion recovery.

The primary objective of this pilot study was to determine whether changes in resting-state FC of the DMN, SN, and CEN differed between adolescents randomized to an app-delivered MBI or app-delivered cognitive sham intervention, from 72 h to 4 weeks post-concussion. The secondary objective was to examine these FC changes in those who met the adherence criteria. As an exploratory objective, we further assessed whether the magnitude of FC changes was greater among participants who adhered to the protocol compared with the full sample, using predefined regions identified in the full sample analysis.

We hypothesized that the adolescents allocated to an MBI, compared to those assigned to a cognitive sham intervention, will exhibit greater alterations in resting-state FC from 72 h to 4 weeks post-concussion in clusters of voxels within cortical regions implicated in the DMN, SN, and CEN. This hypothesis is informed by prior evidence indicating that while MBIs generally increase FC in healthy youth (Jande et al., 2025; Bauer et al., 2020; Bremer et al., 2022; Hafeman et al., 2020; Kennedy et al., 2022; Mishra et al., 2020; Patsenko et al., 2019), acute concussion is characterized by pathological hyperconnectivity in these networks, with reductions in connectivity during recovery (Abbas et al., 2015; Borich et al., 2015; Boshra et al., 2020; Bouchard et al., 2024; Dunkley et al., 2018; Liu et al., 2020; Marusak et al., 2017; Mayer et al., 2011; Newsome et al., 2016; Orr et al., 2016; Zhu et al., 2015). Thus, we expected that MBI may attenuate hyperconnectivity from 72 h to 4 weeks post-concussion. We further anticipated that these effects will be larger in the per-protocol sample than in the intention-to-treat sample, potentially indicating that the observed associations may be amplified with greater adherence to the MBI.

## 2 Methods

### 2.1 Study design

This is an adjunct pilot study to the Health-Canada regulated, randomized, double-blind, placebo-controlled clinical trial “Mindfulness-Based Intervention for Mild Traumatic Brain Injury” (MBI-4-mTBI; Ledoux et al., 2024). The MBI-4-mTBI parent study aimed to evaluate the feasibility and acceptability of implementing an app-delivered MBI for adolescents recovering from acute concussion (Sicard et al., 2026). The intervention was administered via AmDTx, a clinically validated therapeutic mobile platform developed by Mobio Interactive Pte Ltd (Singapore).

The MBI protocol was adapted from the Mindfulness-Based Stress Reduction program (Kabat-Zinn, 1990) structure and content. It included goal setting, psychoeducation, and guided mindfulness exercises (e.g., body scan exercises, mindful breathing; Ledoux et al., 2024; O’Kane et al., 2024; Sicard et al., 2024). Participants in the MBI group were instructed to use the intervention for at least 10 min per day 4 days per week for 4 weeks following enrollment. The sham group received a cognitive sham intervention consisting of arithmetic-based tasks delivered via a similar app and was also instructed to engage with the app at the same frequency and duration as the MBI group.

A neuroimaging pilot study was included as part of MBI-4-mTBI, whereby adolescents with concussion assigned to either the MBI or sham groups underwent magnetic resonance imaging (MRI) at 72 h (±72 h) and 4 weeks (±5 days) post-concussion. The scanning was completed at the Brain Imaging Center of the Royal Ottawa Mental Health Center. The MBI-4-mTBI protocol was approved by the Research Ethics Board of the Children’s Hospital of Eastern Ontario (CHEO). All eligible participants provided either written informed consent or assent with parental consent.

### 2.2 Participants

Adolescents aged 12–17.99 years presenting to the CHEO emergency department or the 360 Concussion Care clinic between February 2023 and June 2024 were enrolled in the MBI-4-mTBI MRI adjunct study if they could attend both MRI sessions, had a physician-diagnosed concussion, scored >4 on the 5P clinical risk score (Zemek et al., 2016), and were proficient in English. Concussions were identified based on the adapted American Congress of Rehabilitation Medicine framework (Silverberg et al., 2023), which included participants with either 1 highest level of certainty symptom or 2 higher level of certainty symptoms immediately or within 1 h of injury. Eligibility criteria for the larger study MBI-4-mTBI are outlined in the published protocol (Ledoux et al., 2024).

Exclusion criteria included a Glasgow Coma Scale score of 13 or lower, abnormal findings on neuroimaging, need for neurosurgical intervention, intubation, or intensive care, and multisystem injuries requiring hospitalization, surgery, or procedural sedation following the intervention. Participants were also excluded if they had severe chronic neurological or developmental conditions affecting communication, were intoxicated at the time of the emergency visit, had no clear history of physical trauma as the primary cause (e.g., seizure, syncope, and migraine), had a prior psychiatric hospitalization, could not provide written consent or assent, lacked a legal guardian, had no internet or mobile/tablet access, or were receiving psychotherapeutic services. Treatment for other medical conditions (e.g., psychiatric medications) or for concussion (e.g., physical therapy) was documented but did not serve as an exclusion criterion. Similarly, prior diagnosis of attention deficit hyperactivity disorder, anxiety, and depression was documented but not exclusionary. Additional, MRI-related exclusion criteria included a history of neurological or neurodevelopmental disorders such as epilepsy, intellectual disability, or autism, a history of stroke or transient ischemic attack, prior sedation medication during the emergency visit, inability to attend both the 72 h and 4 weeks follow-up MRI sessions, or medical contraindications such as claustrophobia, pregnancy, or metal implants (e.g., braces or spacers).

### 2.3 Procedures

The previously published protocol is summarized below (Ledoux et al., 2024). Research assistants recruited participants in the emergency department or concussion clinic. After enrolling in the study, families completed a series of electronic surveys through REDCap (Harris et al., 2009) that captured patient demographics, pre-injury and injury characteristics, symptomatology, and diagnostic history. Measures administered through REDCap included the Health and Behavior Inventory (HBI; Ayr et al., 2009), Pediatric Quality of Life (PedsQL; Varni et al., 2001), Severity Measure for Generalized Anxiety Disorder – Child Age 11–17 (Craske et al., 2013), Center for Epidemiological Studies Depression Scale 10-item version (Andresen et al., 1994), and Child and Adolescent Mindfulness Measure (CAMM; Greco et al., 2011). The day after the ED or concussion clinic visit, participants were called and randomly assigned to either an app-delivered MBI or an app-delivered cognitive sham activity, and to schedule their MRI appointments (72 h and 4-weeks post-injury). At the 4-weeks neuroimaging follow-up, participants completed the cognitive testing using the NIH toolbox (Weintraub et al., 2013). Follow-up and adverse event questionnaires were completed on REDCap at 1, 2, and 4 weeks. Adverse events were defined as an unscheduled emergency department or primary care visit due to exacerbation of symptoms occurring during or within 30 min of app use.

### 2.4 MRI acquisition

Images were acquired on a 3-Tesla Biograph Siemens PET-MRI with a 32-channel head coil. The protocol included a single 12 min 8 s resting-state fMRI sequence as part of the scanning protocol. Participants were instructed to keep their eyes open, fixated on a central crosshair; to avoid movement; and to relax without engaging in any specific thoughts. The resting-state fMRI included the following parameters: resolution = 3.0 mm^3^; slices = 40; field-of-view = 204 mm; repetition time (TR) = 2160 ms; echo time (TE) = 25 ms; flip angle = 70°; acceleration factor = 2; 334 volumes. The high-resolution T1- weighted anatomical imaging used the following parameters: resolution = 1.0 mm^3^; slices = 192; FOV = 256 mm; TR = 2500 ms; TE = 1.69 ms, 3.55 ms, 5.41 ms, and 7.27 ms; flip angle = 7°; inversion time = 1050 ms; phase FOV = 100%; scan time = 5 min 48 s (van der Kouwe et al., 2008). Both the resting-state fMRI and 3D T1-weighted anatomical scans were obtained along the anterior commissure–posterior commissure (AC-PC) line, with full head coverage.

Structural image quality was assessed in accordance with methods established by Backhausen et al. (2016). Preprocessing of fMRI data was performed using a combination of fMRIPrep (Esteban et al., 2019), AFNI (Cox, 1996), and CONN (Whitfield-Gabrieli and Nieto-Castanon, 2012). The fMRIPrep pipeline used tissue segmentation; realignment; susceptibility distortion correction; spatial normalization of structural and functional images to the MNI152 template (2 mm isotropic resolution); and registration of confound regressors. Participant data were excluded if the mean framewise displacement exceeded 0.3 mm, consistent with thresholds used in pediatric imaging (DeSerisy et al., 2020; Frew et al., 2022; Ofoghi et al., 2021; Smith et al., 2022). Spatial smoothing with a full width at half maximum kernel of 6 mm was conducted using the AFNI software, consistent with kernel values reported in fMRI youth research (DeSerisy et al., 2020; Ofoghi et al., 2021). Regressors accounting for global signal, cerebrospinal fluid, white matter, framewise displacement, and motion parameters (rotation and translation x-, y-, and z-axes) were then imported into the CONN toolbox. Global signal was implemented as a regressor to mitigate the effects of non-neuronal noise factors and is consistent with pediatric fMRI research (Aguirre et al., 1997; Fox et al., 2005; Macey et al., 2004; Ofoghi et al., 2021; Power et al., 2017). The CONN denoising procedure incorporated linear regression to remove confounds alongside temporal band-pass filtering (<0.008 Hz or >0.09 Hz). Anatomic labeling of clusters followed the Harvard-Oxford Cortical atlas (Desikan et al., 2006).

### 2.5 Statistical analyses

All research objectives were assessed using both intention-to-treat and per-protocol analyses. The intention-to-treat analyses incorporated all randomized participants, irrespective of their level of adherence to the intervention. In contrast, the per-protocol analyses were limited to participants who met the predefined adherence criterion, engaging in the intervention for at least 10 min per day on a minimum of 4 days per week across a 4-weeks period. Medians and interquartile ranges were calculated using available data for clinical measures at baseline and follow-up within both the intention-to-treat and per-protocol samples to characterize each sample. Adherence to the study protocol was measured using device-recorded engagement data, specifically the duration of active participation in the assigned intervention, recorded in seconds.

Independent *t*-tests and chi-square tests were used to assess group differences in demographic data across timepoints, with statistical significance set at α = 0.05. Scores exceeding 2.48 standard deviations from the mean were classified as outliers (Cousineau and Chartier, 2010). Missing behavioral data were addressed using expectation maximization (Dong and Peng, 2013).

The research objectives were analyzed with the following regression model:


$$FC_{ij}=\beta 0+\beta 1(Group_{j})+\beta 2(Time_{i})+\beta 3(Group_{j}\times Time_{i})+\text{ε}$$

where *FC*_*ij*_ represents the functional connectivity (FC) between the specified seed and each voxel across the brain for participant *i* at time point *j*. *β_0_* is the intercept term. *β1(Group_*j*_)* is a binary variable representing the group (MBI or Sham) at time point *j*. *β2(Time_*i*_)* is a binary variable representing the effects of time for participant *i*. *β3(Group_*j*_* × *Time_*i*_)* represents the interaction effect between group and time, the key term for all reported results. ε represents the error term.

Region of interest (ROI) analyses were used to assess FC patterns between DMN, SN, and CEN seeds. The selected ROIs were organized using hierarchical clustering, applying the CONN toolbox’s default weighting (0.05) to account for either anatomical closeness (1 = proximal) or functional similarity (0 = functional). Clusters were considered significant if they met a connection-level threshold of *p* < 0.05 (uncorrected) and a cluster-level threshold of *p* < 0.05, corrected for FDR across all ROI-to-ROI connections in the analysis. A significant cluster refers to a consistent FC pattern among a group of ROI connections, even if individual connections within the cluster are not independently significant.

Seed-to-voxel analyses were used to assess FC between *a priori*, CONN-defined seeds from the DMN, SN, and CEN with every voxel in the brain. Seed-based connectivity maps were generated to measure FC between each network seed and the rest of brain. The selection of these network seeds was grounded in their reported involvement in concussion (Abbas et al., 2015; Borich et al., 2015; Iyer et al., 2019; Johnson et al., 2012; Mayer et al., 2011; Meier et al., 2017; Plourde et al., 2021; Sours et al., 2013, 2015; van der Horn et al., 2023) and mindfulness (Bremer et al., 2022; Brewer et al., 2011; Doll et al., 2015; Farb et al., 2007; Taylor et al., 2013; Zhang et al., 2023). The DMN seeds included the medial prefrontal cortex, left- and right-side lateral parietal cortex, and posterior cingulate cortex. The CEN seeds included the left- and right-side lateral prefrontal cortex, as well as the left- and right-side posterior parietal cortex. The SN seeds included the anterior cingulate cortex, left- and right-side anterior insula, left- and right-side rostral prefrontal cortex, as well as the left- and right-side supramarginal gyrus. Connectivity maps were generated using Random Field Theory cluster inference (Worsley et al., 1996), with an applied voxel-level height threshold of *p* < 0.001 (uncorrected) and a cluster-level threshold at *p* < 0.050, corrected for false discovery rate (FDR).

In addition to the primary and secondary objective analyses, we conducted an exploratory per-protocol analysis to examine whether the magnitude of FC changes differed among participants who adhered to the intervention (per-protocol) relative to the full sample (intention-to-treat). Given the smaller sample size, we anticipated reduced power to detect significant clusters after multiple comparison correction. To explore potential trends, significant Group × Time interaction clusters identified in the intention-to-treat seed-to-voxel analysis were used as masks. The identical second-level model was then re-estimated in the per-protocol sample, and both unstandardized β coefficients and standardized effect sizes were extracted from these regions. Standardized effect sizes were derived by converting the cluster-level *F*-values to partial correlation coefficients. These results are presented descriptively as an exploratory comparison of effect magnitude between intention-to-treat and per-protocol samples, without additional inference testing.

## 3 Results

Thirty-six participants were included in the intention-to-treat approach for this study (see Figure 1 flow chart). The MBI group consisted of 16 participants (31% female, age median [IQR]: 14.23 [13.52–15.65]). The sham group consisted of 20 participants (45% female, age median [IQR]: 14.05 [13.13–16.26]). Sport was the most common mechanism of injury for both groups (MBI: 12/16 [75%]; Sham: 14/20 [70%]). There were no significant differences between groups for age, sex, diagnostic history (depression, anxiety, and attention deficit hyperactivity disorder), or concussion history. Adherence was comparable between groups, with similar minutes of active participation in the intervention (MBI: median = 214.39 min, IQR = 85.70–324.02 min; Sham: median = 183.47 min, IQR = 84.64–495.74 min, *p* = 0.586). Table 1 presents demographic results.

**FIGURE 1:**
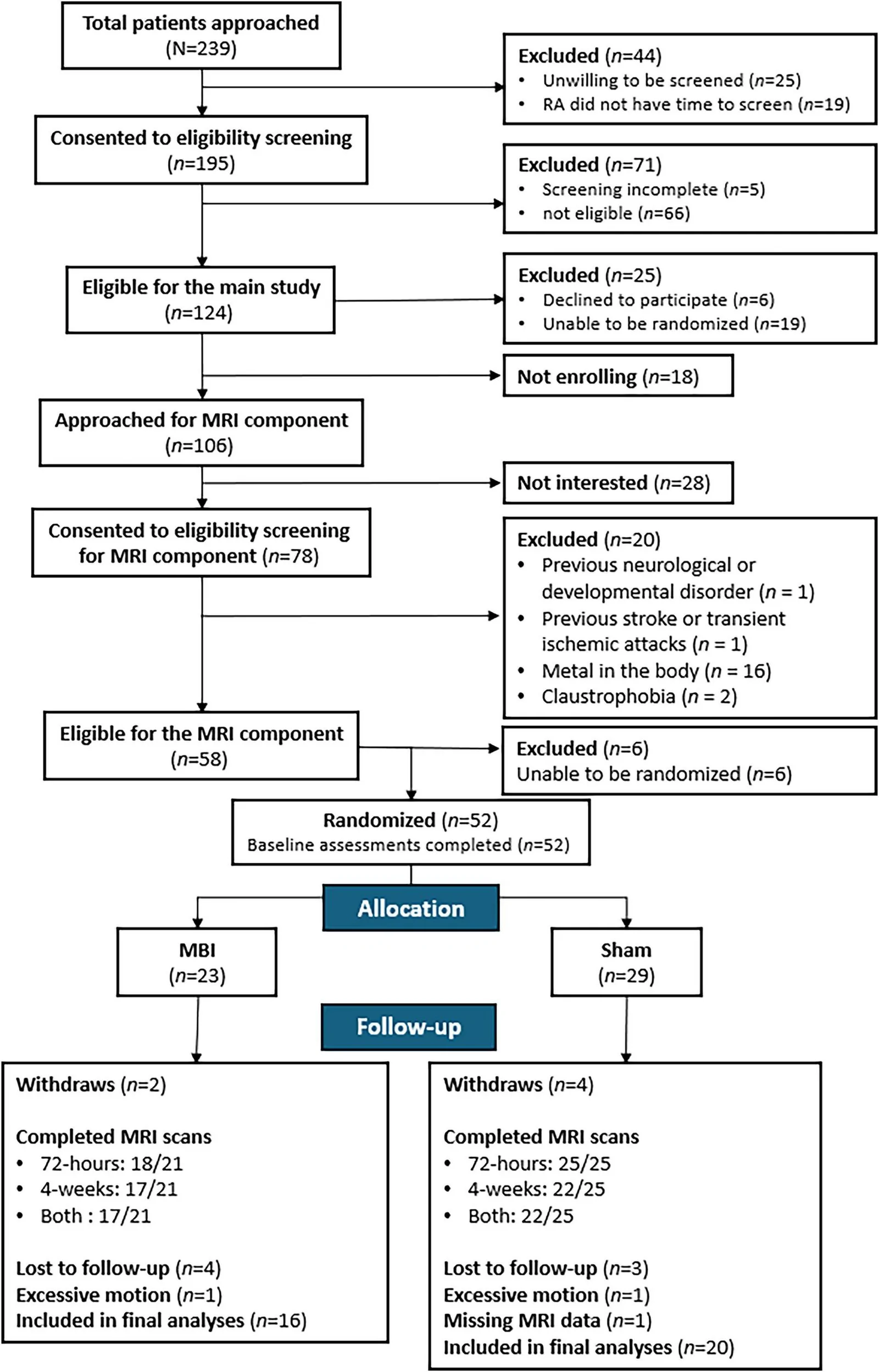
Illustration of participant enrolment, randomization, and completion information.

**TABLE 1:** Demographics.

Variables	Intention-to-treat MBI *n* = 16	Intention-to-treat Sham *n* = 20	Intention-to-treat comparison *p*	Per-protocol MBI *n* = 10	Per-protocol Sham *n* = 13	Per-protocol comparison *p*
Age | *Mdn* (IQR)	14.23 (13.52–15.65)	14.05 (13.13–16.26)	0.815	14.90 (14.01–15.66)	14.66 (13.61–16.59)	0.496
Sex | *n* (%)			0.427			0.811
Males	11 (69%)	11 (55%)		6 (60%)	7 (54%)	
Female	5 (31%)	9 (45%)		4 (40%)	6 (46%)	
Diagnostic History | *n* (%)			0.818			0.878
Depression	1 (6%)	0		1 (10%)	0	
Anxiety	1 (6%)	3 (15%)		0	3 (23%)	
Prior concussion | *n* (%)	6 (38%)	9 (45%)	0.910	3 (30%)	8 (62%)	0.280
Mechanism of injury | *n* (%)			0.156			0.455
Sport	12 (75%)	14 (70%)		7 (70%)	9 (69%)	
Fall from standing/walking/ running	0	4 (20%)		0	2 (15%)	
Other mechanism	3 (19%)	2 (10%)		2 (20%)	2 (15%)	

MBI, mindfulness-based intervention.

Twenty-three participants were included as part of the per-protocol approach for this study, having adhered to the intervention. The MBI group consisted of 10 participants (40% female, age: 14.90 [14.01–15.66]). The sham group consisted of 13 participants (46% female, age: 14.66 [13.61–16.59]). No significant between-group differences were observed for age, sex, diagnostic history, or concussion history. Adherence was comparable between groups (MBI: median = 300.65 min, IQR = 225.45–339.22 min; Sham: median = 340.78 min, IQR = 193.88–636.62 min, *p* = 0.495). Descriptive clinical outcome measures at baseline and follow-up are presented in Tables 2, 3. No adverse events were reported by any participant in either group.

**TABLE 2:** Clinical outcome measures, intention-to-treat.

	Baseline				Follow-up			
Variables	MBI	*n*	Sham	*n*	MBI	*n*	Sham	*n*
HBI | *Mdn* (IQR)	23.00 (13.00, 32.25)	16	17.00 (11.50, 27.00)	20	11.50 (9.25, 20.50)	16	12.00 (5.00, 16.00)	19
PedsQL | *Mdn* (IQR)	78.26 (70.11, 89.67)	15	84.78 (72.28, 91.84)	19	84.78 (69.84, 87.77)	16	86.96 (77.17, 96.20)	19
GAD | *Mdn* (IQR)	4.00 (1.00, 14.00)	15	1.00 (0.00, 10.50)	19	3.00 (1.50, 8.75)	16	3.00 (0.00, 9.50)	19
CES-D-10 | *Mdn* (IQR)	4.00 (3.50, 8.50)	15	4.00 (3.00, 6.00)	19	6.50 (5.25, 10.00)	16	4.00 (2.50, 6.00)	19
CAMM | *Mdn* (IQR)	33.00 (26.50, 36.00)	15	33.00 (20.50, 36.00)	19	35.50 (28.25, 38.25)	16	32.00 (24.50, 37.50)	19
NIH Toolbox Fluid Composite Score | *Mdn* (IQR)					106.00 (90.50, 117.00)	15	107.00 (99.25, 120.00)	18

MBI, mindfulness-based intervention; HBI, Health and Behavior Inventory; PedsQL, Pediatric Quality of Life Inventor; GAD, Severity Measure for Generalized Anxiety Disorder – Child Age 11–17; CES-D-10, Center for Epidemiologic Studies Depression Scale-10; CAMM, Child and Adolescent Mindfulness Measure; NIH Toolbox Fluid Composite Score represents the age-corrected standard score. Medians and interquartile ranges are based on available data.

**TABLE 3:** Clinical outcome measures, per-protocol.

	Baseline				Follow-up			
Variables	MBI	*n*	Sham	*n*	MBI	*n*	Sham	*n*
HBI | *Mdn* (IQR)	23.00 (13.50, 30.25)	10	17.00 (13.00, 26.00)	13	15.00 (7.75, 21.50)	10	12.00 (9.00, 15.00)	13
PedsQL | *Mdn* (IQR)	78.26 (63.04, 88.04)	9	79.35 (70.65, 86.68)	12	86.41 (63.86, 89.40)	10	83.70 (77.17, 86.96)	13
GAD | *Mdn* (IQR)	8.00 (3.00, 14.00)	9	6.00 (1.00, 12.50)	12	5.00 (2.00, 10.25)	10	8.00 (0.00, 10.00)	13
CES-D-10 | *Mdn* (IQR)	5.00 (4.00, 12.00)	9	4.50 (3.00, 7.50)	12	7.50 (6.00, 10.75)	10	5.00 (4.00, 6.00)	13
CAMM | *Mdn* (IQR)	31.00 (20.00, 33.00)	9	27.50 (18.00, 34.50)	12	33.50 (21.50, 36.75)	10	31.00 (23.00, 36.75)	13
NIH Toolbox Fluid Composite Score | *Mdn* (IQR)					114.00 (98.00, 127.25)	10	110.00 (101.00, 123.00)	12

MBI, mindfulness-based intervention; HBI, Health and Behavior Inventory; PedsQL, Pediatric Quality of Life Inventor; GAD, Severity Measure for Generalized Anxiety Disorder – Child Age 11–17; CES-D-10, Center for Epidemiologic Studies Depression Scale-10; CAMM, Child and Adolescent Mindfulness Measure; NIH Toolbox Fluid Composite Score represents the age-corrected standard score. Medians and interquartile ranges are based on available data.

### 3.1 Intention-to-treat, ROI-to-ROI

A significant cluster was detected, comprising connections between DMN and CEN nodes. Specifically, the identified cluster comprised three cortical regions: the medial prefrontal cortex, left posterior parietal cortex, and left lateral prefrontal cortex (cluster *F*[2,33] = 6.51, FDR corrected *p* = 0.025; Figure 2), with two connections among them: one between the medial prefrontal cortex and the left posterior parietal cortex (MBI FC β = −0.049; Sham FC β = 0.039), and another between the medial prefrontal cortex and the left lateral prefrontal cortex (MBI FC β = −0.05; Sham FC β = 0.047).

**FIGURE 2:**
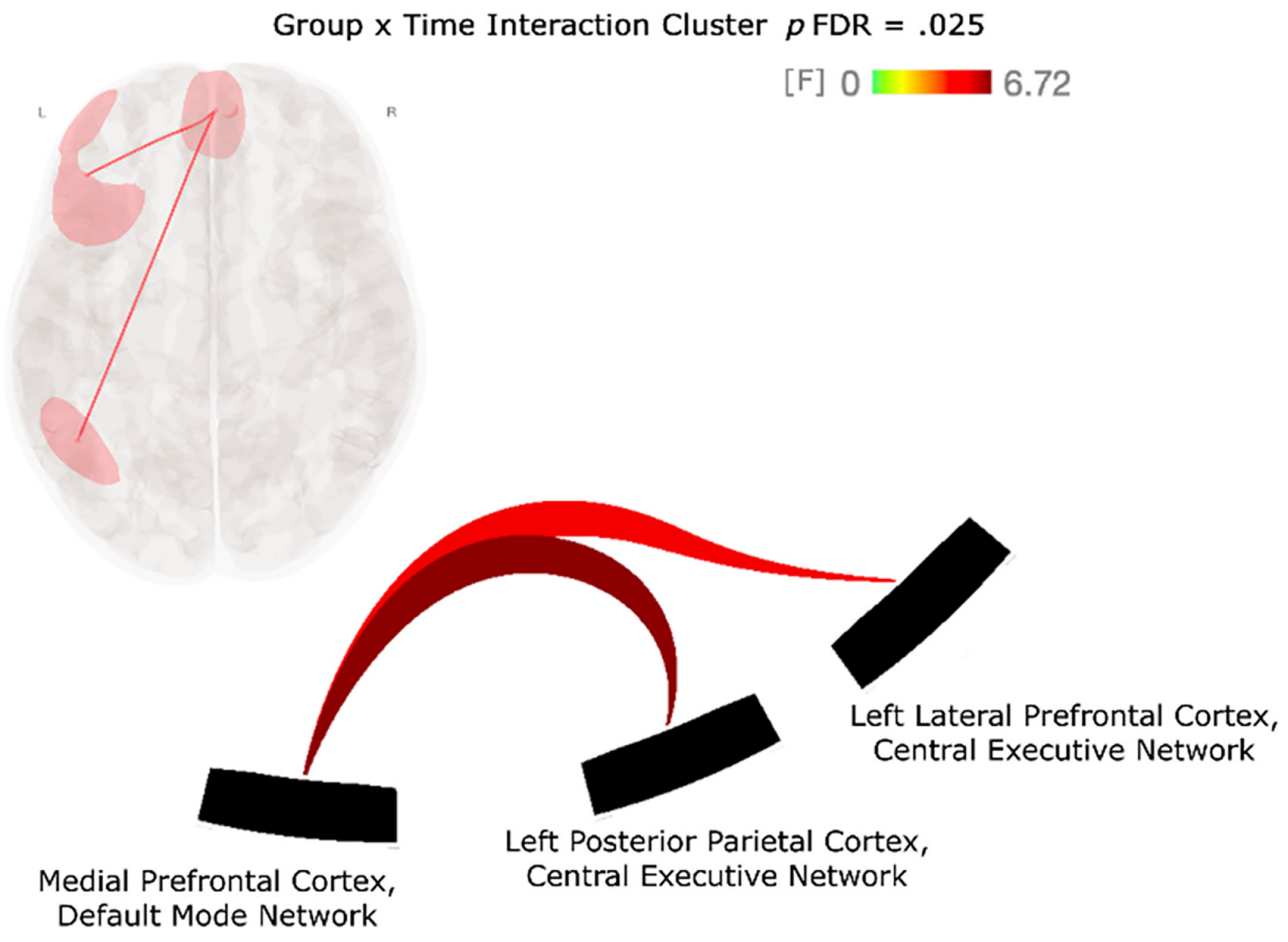
A significant group-by-time interaction, whereby patterns of default mode network (DMN) to central executive network (CEN) functional connectivity (FC) from 72 h to 4-weeks post-injury differed significantly between mindfulness-based intervention (MBI) and sham groups. Specifically, patterns of DMN-CEN FC differed in a cluster of nodes including the medial prefrontal cortex, left posterior parietal cortex, and left lateral prefrontal cortex, whereby FC between these nodes decreased in the MBI group and increased in the sham group. The color scale represents *F*-values for each connection of the significant cluster.

### 3.2 Per-protocol, ROI-to-ROI

No significant clusters were identified in the per-protocol ROI-to-ROI analyses.

### 3.3 Intention-to-treat, seed-to-voxel

Four significant clusters were identified using DMN seeds. These reflected the interaction between Group and Time on DMN FC (Table 4). Significant interactions were observed between the medial prefrontal cortex and the anterior portion of the left superior frontal gyrus (SFG; Figure 3a), as well as between the medial PFC and a more posterior region of the left SFG (Figure 3b), wherein the MBI group showed a greater decrease in FC over time compared to the sham group. Significant interactions were observed between the medial prefrontal cortex and the right inferior temporal gyrus (Figure 3c), as well as between the left lateral parietal cortex and the right superior frontal gyrus (Figure 3d), wherein the MBI group showed a greater increase in FC over time compared to the sham group.

**TABLE 4:** Significant interaction clusters in the intention-to-treat analysis.

Seed, *Network*	Cluster label	Cluster size	MNI coordinates (x, y, z)	*F*[1,34]	FDR-*p*	MBI β	Sham β
Medial prefrontal cortex, *DMN*	Left superior frontal gyrus	140	−08, +38, +38	34.36	0.001	−0.069	0.053
Medial prefrontal cortex, *DMN*	Left superior frontal gyrus	79	−08, +32, +54	29.94	0.020	−0.074	0.052
Medial prefrontal cortex, *DMN*	Right inferior temporal gyrus	60	+50, −16, −32	25.84	0.047	0.061	−0.059
Left lateral parietal, *DMN*	Right superior frontal gyrus	91	+04, +52, +36	28.13	0.020	−0.067	0.056
Right supramarginal gyrus, *SN*	Left superior frontal gyrus	74	−20, +18, +64	25.06	0.040	0.071	−0.059
Left posterior parietal, *CEN*	Left frontal pole	77	−24, +44, +40	28.17	0.024	−0.052	0.056

CEN, central executive network; DMN, default mode network; MBI, mindfulness-based intervention; SN, salience network. Cluster size refers to the number of voxels based on dimensions of the MNI standard space (2 mm^3^).

**FIGURE 3:**
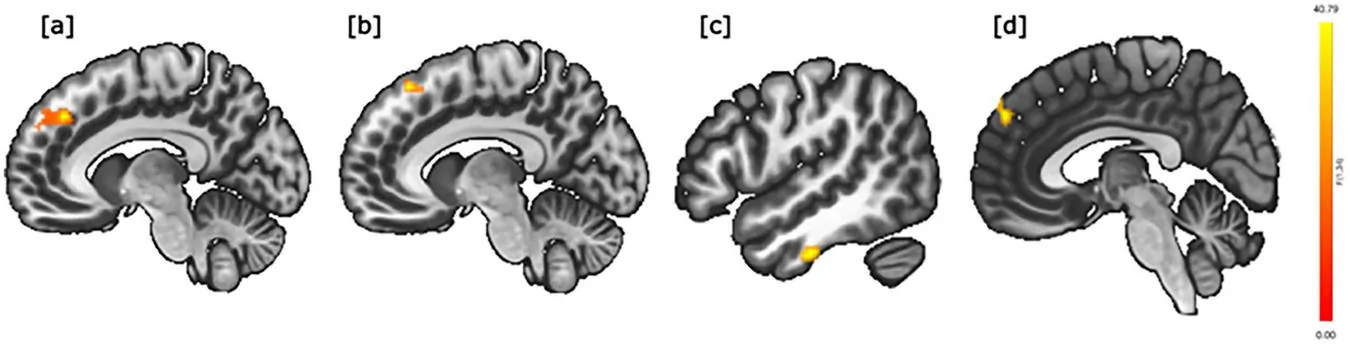
Sagittal brain slices overlaid on an MNI template, highlighting significant clusters reflecting the interaction between group and time on default mode network (DMN) functional connectivity (FC). **(a)** Interaction between the medial prefrontal cortex seed and left superior frontal gyrus, where the mindfulness-based intervention (MBI) group showed a greater decrease in FC over time compared to the sham group. **(b)** Interaction between the medial prefrontal cortex seed and left superior frontal gyrus, where the MBI group showed a greater decrease in FC over time compared to the sham group. **(c)** Interaction between the medial prefrontal cortex seed and right inferior temporal gyrus, where the MBI group showed a greater increase in FC over time compared to the sham group. **(d)** Interaction between the left lateral parietal cortex seed and right superior frontal gyrus, where the MBI group showed a greater increase in FC over time compared to the sham group. The heatmaps represent *F*-values for each cluster.

A significant cluster was identified using an SN seed, reflecting the interaction between group and time on SN FC (Table 4). The significant interaction was observed between the right supramarginal gyrus seed and the left superior frontal gyrus, where the MBI group showed a greater increase in FC from 72 h to 4 weeks compared to the sham group (Figure 4a).

**FIGURE 4:**
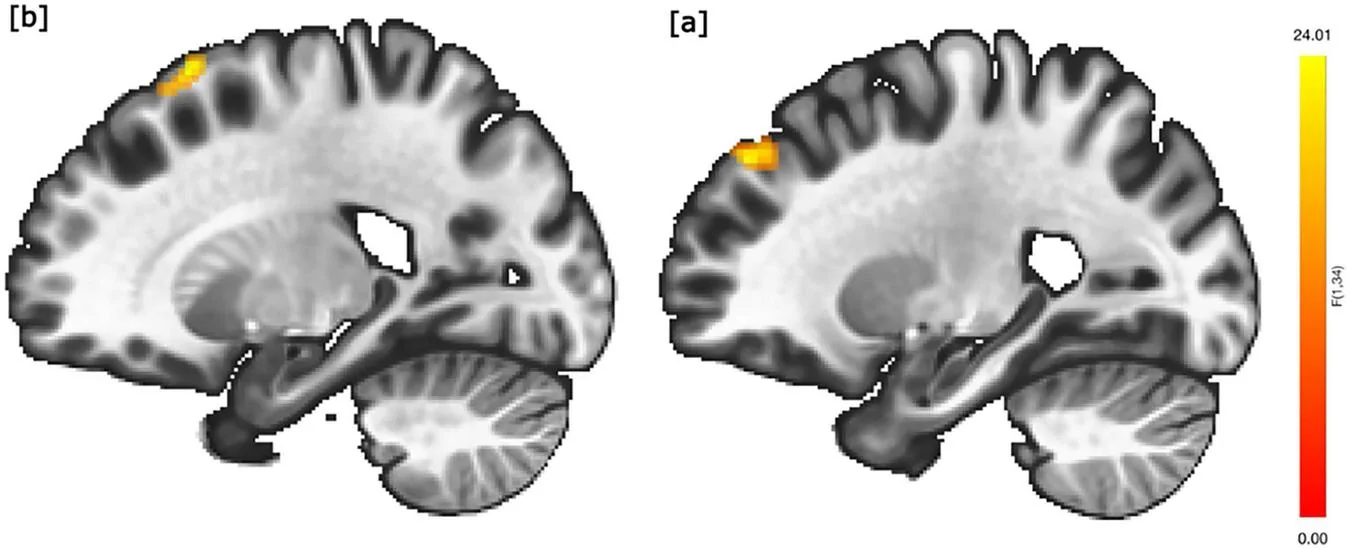
Sagittal brain slice overlaid on an MNI template, highlighting significant clusters reflecting the interaction between group and time on functional connectivity (FC). **(a)** The interaction was observed between the right supramarginal gyrus seed and the left superior frontal gyrus, where the MBI group showed a greater increase in FC from 72 h to 4 weeks compared to the sham group. **(b)** The interaction was observed between the left posterior parietal cortex seed and the left frontal pole, where the sham group exhibited a greater increase in FC from 72 h to 4 weeks compared to the MBI group. The heatmap represents *F*-values for the cluster.

A significant cluster was also identified using a CEN seed, reflecting the interaction between group and time on CEN FC (Table 4). The significant interaction was observed between the left posterior parietal cortex seed and the left frontal pole, where the sham group exhibited a greater increase in FC from 72 h to 4 weeks compared to the MBI group (Figure 4b).

### 3.4 Per-protocol, seed-to-voxel

A significant cluster was identified using the medial prefrontal cortex seed of the DMN, reflecting the interaction between group and time on DMN FC. The significant interaction was observed between the medial prefrontal cortex seed and left intracalcarine cortex, where the sham group showed a greater decrease in FC over time compared to the MBI group (cluster size = 190, MNI x y z = −06 −78 +08, *F*[1, 21] = 32.39, FDR corrected *p* < 0.001; MBI β = 0.124, Sham β = −0.103; Figure 5).

**FIGURE 5:**
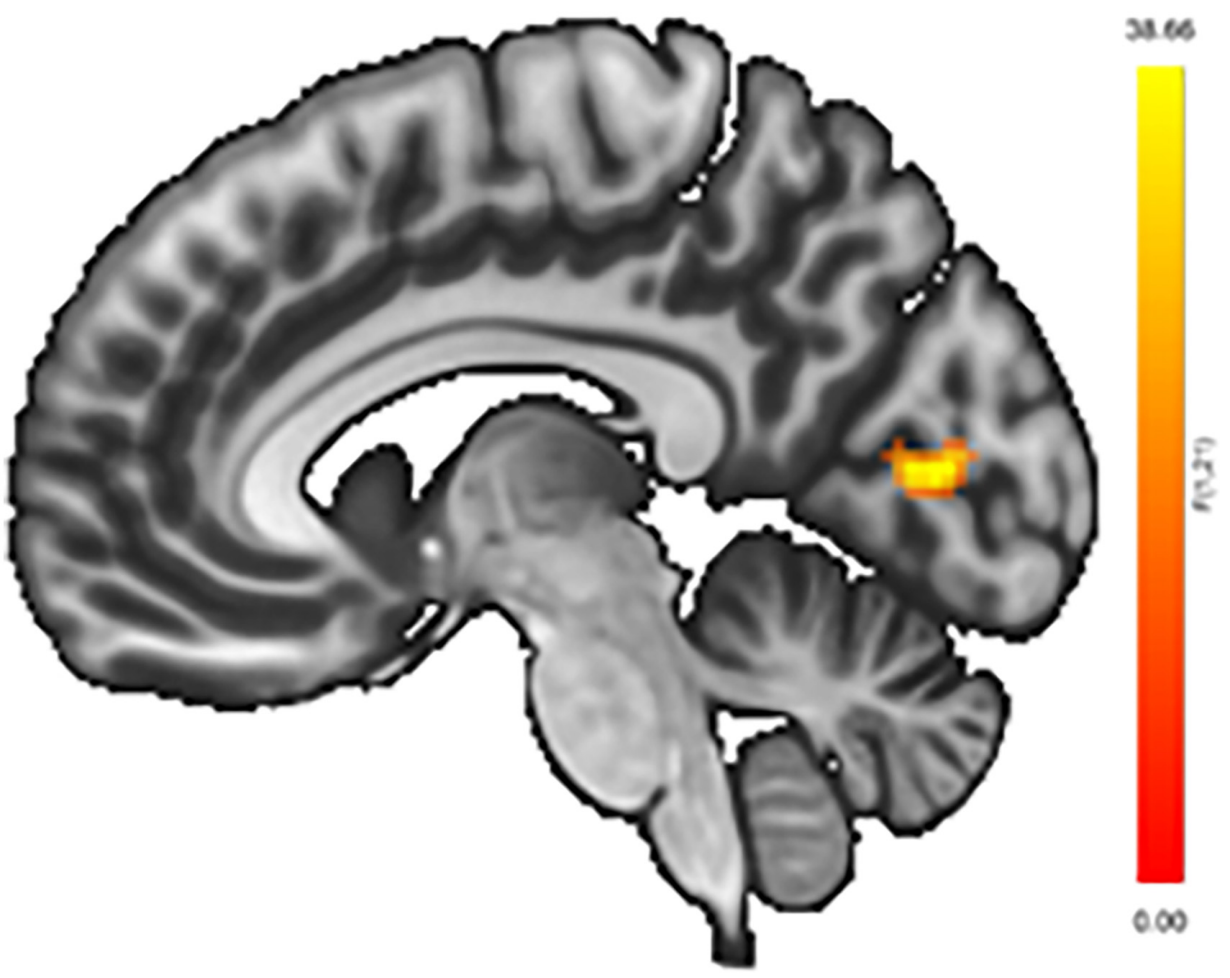
Sagittal brain slice overlaid on an MNI template, highlighting a significant cluster reflecting the interaction between group and time on default mode network (DMN) functional connectivity (FC). The interaction was observed between the medial prefrontal cortex seed and the left intracalcarine cortex, where the sham group showed a greater decrease in FC from 72 h to 4 weeks compared to the MBI group. The heatmap represents *F*-values for the cluster.

### 3.5 Exploratory analysis

Across the identified seed-to-voxel clusters, effect sizes (partial correlation coefficients) in the per-protocol sample were generally similar to or slightly larger in magnitude than those observed in the intention-to-treat sample (Table 5). In some regions, the per-protocol effect sizes were descriptively further from zero, suggesting a trend toward stronger FC changes among participants who adhered to the intervention. However, because these clusters were identified in the intention-to-treat analysis and did not reach statistical significance in the per-protocol sample, no formal statistical comparison of effect sizes was performed.

**TABLE 5:** Effect sizes for intention-to-treat derived clusters in intention-to-treat and per-protocol samples.

Seed, *Network*	Cluster label	Cluster size	MNI coordinates (x, y, z)	*r*_*partial*_	*r*_*partial*_
Medial prefrontal cortex, *DMN*	Left superior frontal gyrus	140	−08, +38, +38	−0.709	−0.722
Medial prefrontal cortex, *DMN*	Left superior frontal gyrus	79	−08, +32, +54	−0.684	−0.730
Medial prefrontal cortex, *DMN*	Right inferior temporal gyrus	60	+50, −16, −32	0.657	0.646
Left lateral parietal, *DMN*	Right superior frontal gyrus	91	+04, +52, +36	−0.673	−0.585
Right supramarginal gyrus, *SN*	Left superior frontal gyrus	74	−20, +18, +64	0.651	0.688
Left posterior parietal, *CEN*	Left frontal pole	77	−24, +44, +40	−0.673	0.640

CEN, central executive network; DMN, default mode network; MBI, mindfulness-based intervention; SN, salience network.

## 4 Discussion

This study examined the effects of an app-delivered MBI on resting-state FC of three major neural networks: DMN, SN, and CEN in adolescents recovering from concussion. Our findings indicate that MBI influences FC patterns over time, evidenced by significant group-by-time interactions, suggesting that mindfulness training might reflect neurofunctional recovery mechanisms in pediatric concussion.

The ROI-to-ROI analysis identified a significant group-by-time interaction involving FC patterns between DMN and CEN regions. Specifically, FC decreased between the medial prefrontal cortex (DMN node) and both the left posterior parietal cortex and left lateral prefrontal cortex (CEN nodes) in the MBI group, whereas these connections increased in the sham group. These regions represent nodes of the DMN and CEN, respectively, and their altered patterns of connectivity, which may indicate a restoration of functional segregation (i.e., anticorrelation) between networks following mindfulness training (Bauer et al., 2020). This is consistent with recent findings that MBI preserved anticorrelation between the DMN and dorsolateral prefrontal cortex (Bauer et al., 2020). Reduced DMN-CEN FC (i.e., a more segregated network system) in the MBI group also aligns with previous research suggesting that hyperconnectivity between these networks reflects maladaptive compensatory responses after concussion (Mayer et al., 2011; Hillary et al., 2015). Hyperconnectivity typically refers to excessive communication between brain regions or networks that are normally more functionally segregated. This pattern is often interpreted as an early compensatory mechanism, whereby the brain recruits additional or redundant neural resources to maintain performance in the face of injury (Giza and Hovda, 2014; Mayer et al., 2011; Hillary et al., 2015). While such compensatory recruitment may be typical in the acute phase of injury, persistent hyperconnectivity beyond the initial recovery window is thought to become maladaptive, potentially reflecting inefficient network dynamics, cognitive fatigue, and interference between competing processes (Hillary et al., 2015; Mayer et al., 2011). Our study, conducted within 72 h of injury, offers a unique window into these early neural responses. It is possible that the reduced DMN–CEN FC observed in the MBI group reflects an early moderation of this hyperconnectivity, potentially influencing network activity toward a more efficient configuration before maladaptive patterns consolidate.

The seed-to-voxel analyses identified four significant clusters of DMN FC differences over time. In support of our hypothesis, the MBI group, relative to the sham group, showed greater decreases in FC between three DMN nodes and the superior frontal gyrus (SFG). This included the medial prefrontal cortex and two clusters along the left dorsomedial and dorsolateral regions of the SFG, respectively. The MBI group also showed a decrease in FC between the left lateral parietal cortex and a cluster along the right dorsomedial region of the SFG. SFG subregions have specific connectivity patterns implicated in task-positive networks, such as the CEN, as well as the DMN (Li et al., 2013). A parcellation report suggests that anteromedial SFG is linked to cognitive control and default mode networks, while the dorsolateral SFG is linked to the CEN (Li et al., 2013). A previous study of healthy youth suggested that greater connectivity between DMN and SFG was associated with greater pain frequency in adolescents, which was thought to reflect less segregation of DMN and CEN activity (Jones et al., 2020). Another fMRI study found that MBI preserved anticorrelation of function between the DMN and dorsolateral prefrontal cortex, a key node of the CEN (Bauer et al., 2020). These findings corroborate our DMN-CEN finding detected in the ROI analysis and might suggest that reduced FC between DMN nodes and the SFG potentially reflect attenuated hyperconnectivity and preservation of anticorrelation between DMN and CEN. By reducing excessive FC in circuits where hyperconnectivity might be inefficient or compensatory, while simultaneously strengthening connections that support adaptive integration, MBIs might promote more efficient network functioning.

The MBI group also showed a greater increase in FC between the medial prefrontal cortex (DMN node) and the right inferior temporal gyrus. Taken together with the reductions in DMN–CEN and DMN–SFG connectivity, this pattern highlights that MBI-related network changes are not uniformly directional. Instead, MBIs may simultaneously dampen excessive connectivity in circuits where hyperconnectivity is maladaptive, while strengthening connectivity in pathways that support adaptive integration. This bidirectional moderation suggests that MBI effects might be network- and function-specific, rather than simply increasing or decreasing overall connectivity. This increase in FC might thus reflect an adaptive recovery of brain function consistent with patterns observed in mindfulness practitioners (Hillary et al., 2015; Sharp et al., 2014; Farb et al., 2007). This might also reflect enhanced integration of internal thought processes, suggesting more adaptive present-moment awareness with attentional and emotional regulation benefits attributed to mindfulness (Bauer et al., 2020; Hafeman et al., 2020; Mishra et al., 2020; Patsenko et al., 2019).

Concussion research suggests that SN FC and its ability to moderate network switching are frequently disrupted following traumatic brain injury (Menon, 2011; Jilka et al., 2014), potentially associated with increased FC within the DMN and hyperconnectivity of the DMN and CEN in adolescents (Mayer et al., 2011). In this study, the MBI group, relative to the sham group, exhibited a greater increase in FC between the right supramarginal gyrus and the left SFG. Although concussion research reports increased FC within the SN between the frontoinsular cortex and dorsal anterior cingulate cortex at 1 month post-injury (Healey et al., 2022), the increased SN FC between the supramarginal gyrus and SFG reported here is consistent with a recent study suggesting that mindfulness practices might enhance SN function by improving FC between the SN and regions of the CEN (Bremer et al., 2022). As such, the relative increase in FC between the SN and SFG in the MBI group might mitigate the effects of DMN and CEN hyperconnectivity following concussion. As the SN, particularly the supramarginal gyrus, is implicated in attention allocation and salience detection, increased supramarginal gyrus-SFG FC might be associated with improved attentional control, emotional regulation, and task readiness. Future research should investigate whether these connectivity changes are associated with clinical outcomes in a larger sample.

Studies of healthy adults have reported that increased SN connectivity following MBI is associated with improved attentional focus and emotional regulation (Zeidan et al., 2011; Farb et al., 2007). Given the SN’s proposed role in switching between the DMN and CEN, the reported FC differences may indicate cognitive and emotional processes in the MBI group (Tang, 2017). Moreover, a positive correlation between mindfulness scores and intrinsic FC of the SN and CEN suggests that increased FC may reflect neural mechanisms of mindfulness (Doll et al., 2015). MBI could thus promote neurofunctional recovery by enhancing SN function following pediatric concussion, though further research is required to explore this possibility.

The sham group showed a greater FC increase between the left posterior parietal cortex and the left frontal pole when compared to the MBI group. This finding is notable given the frontal pole’s role in both CEN and DMN function, as it might serve as a moderator of activity between these networks (Spreng et al., 2010).

Although MBI studies have reported increased FC of the CEN (Jande et al., 2025), the decreased FC between CEN and the frontal pole in the MBI group may instead reflect a reduction in the pathological hyperconnectivity commonly observed following concussion (Mayer et al., 2011). Such hyperconnectivity is thought to represent compensatory but inefficient neural recruitment patterns in response to injury (Hillary et al., 2015). The MBI-related attenuation of this connectivity pattern suggests that MBI might help restore more efficient network functioning. This potential normalization may support improved attentional control and executive functioning, as the frontal pole becomes less influenced by competing network demands. These findings align with the notion that the positive effects of MBI on behavioral outcomes are likely mediated by functional reorganization within and between the DMN, SN, and CEN (Jande et al., 2025; Melis et al., 2022; Young et al., 2018).

While the reduced statistical power in the per-protocol sample limited the probability of detecting true significant clusters after multiple comparison corrections, the exploratory analyses indicated that the magnitude of FC changes in the per-protocol sample was greater than that in the intention-to-treat sample in several clusters. This may suggest that greater adherence to MBI enhances its effects on some neural connections; however, it is also possible that personality features of participants who adhere contributed to these differences. This supports previous reports that increased MBI engagement is linked to greater behavioral benefits, though few published randomized controlled trials explicitly investigated dose-response relationships in MBI [see Creswell (2017) for a review]. Research examining the impact of adherence on MBI-related outcomes is warranted.

This study has several notable strengths. It employed a pragmatic randomized controlled trial design, following the Consolidated Standards of Reporting Trials (CONSORT) framework (Hopewell et al., 2025). The custom, app-delivered MBI was novel, feasible, and acceptable for adolescent participants, with engagement objectively tracked through device-based measures of app usage (Sicard et al., 2024). Importantly, the primary outcomes were derived from neuroimaging metrics, providing objective measures of resting-state network connectivity and further reducing reliance on subjective reporting.

While numerous significant clusters were identified, it is important to note that this is a substudy of a feasibility trial. As a result, the smaller sample size inherently limits the statistical power and generalizability of findings. Although small samples may risk spurious results for brain-wide exploratory analyses (Marek et al., 2022), the present study employed an *a priori* hypothesis-driven approach using predefined networks and seed-based connectivity to reduce analytic complexity for pilot neuroimaging research. Future research should also implement additional timepoints during the chronic phase of injury (e.g., >6 months post-concussion) to examine the effects of extending the MBI. Methodological considerations might also influence outcomes, particularly regarding our seed selection approach. The specific anatomical locations chosen for seed-to-voxel analyses can substantially alter network connectivity patterns and subsequent interpretations (Buckner et al., 2008; Cole et al., 2010; Hayasaka and Laurienti, 2010). Replication studies using comparable seed coordinates are essential to validate our results. Furthermore, additional investigations using unique advanced imaging techniques, such as arterial spin labeling, diffusion tensor imaging, and magnetic resonance spectroscopy, are recommended to establish a better understanding of the neurofunctional associations reported in this study. Nonetheless, the small sample size remains a limitation, and replication in larger cohorts is required to confirm the reliability and reproducibility of these targeted network-level effects. Larger trials over extended periods with multimodal neuroimaging approaches are thus needed to validate and extend these findings.

Although descriptive clinical outcome measures were collected for the MRI substudy, this imaging sample was not designed to examine the relationship between longitudinal changes in functional connectivity and changes in patient-reported outcomes. Further, the study sample is not powered to determine clinical efficacy. Descriptively, both treatment groups demonstrated broadly similar improvement over time, consistent with the expected clinical recovery trajectory following pediatric concussion (Yeates et al., 2025). Previous work has shown that neuroimaging abnormalities may persist despite clinical recovery (Polak et al., 2015), suggesting that imaging and symptom recovery do not necessarily occur in parallel. Future adequately powered MBI studies should jointly model patient-reported outcomes and neuroimaging measures to determine whether functional connectivity changes moderate or mediate clinical recovery.

## 5 Conclusion

This study found that an MBI influenced resting-state FC in neural networks implicated in pediatric concussion recovery. Specifically, we found that MBI is associated with changes in key brain networks involved in attention, emotional regulation, and cognitive function, though our findings do not provide evidence that these neural changes directly translated into measurable behavioral improvements in this feasibility substudy. This study provides preliminary evidence supporting further evaluation of app-delivered MBI as a potential adjunctive treatment for pediatric concussion. If additional studies corroborate these findings, MBI delivered via app could become a feasible, accessible, scalable, and empowering tool for adolescents managing symptoms after a concussion.

## Data Availability

The data cannot be shared due to legal, ethical or privacy restrictions. Requests to access the datasets should be directed to A-AL, aledoux@cheo.on.ca.
